# Methanol Extract of Aerial Parts of *Pavetta indica* L. Enhances the Cytotoxic Effect of Doxorubicin and Induces Radiation Sensitization in MDA-MB-231 Triple-Negative Breast Cancer Cells

**DOI:** 10.3390/molecules24122273

**Published:** 2019-06-18

**Authors:** Yen Thi-Kim Nguyen, Jeong Yong Moon, Ji-yeon Ryu, Sangmi Eum, Tran The Bach, Somi Kim Cho

**Affiliations:** 1 Interdisciplinary Graduate Program in Advanced Convergence Technology and Science, Jeju National University, Jeju 63243, Korea; ntkyen.hcmus@gmail.com; 2 Subtropical/tropical organism gene bank, Jeju National University, Jeju 63243, Korea; owenmjy@jejunu.ac.kr; 3 School of Biomaterials Sciences and Technology, College of Applied Life Sciences, SARI, Jeju National University, Jeju 63243, Korea; rjo211@naver.com; 4 International Biological Material Research Center, Korea Research Institute of Bioscience & Biotechnology, 125, Gwahak-ro, Yuseong-gu, Daejeon 34141, Korea; sangeum@gmail.com; 5 Institute of Ecology and Biological Resources, Vietnam Academy of Science and Technology (VAST), Ha Noi 10000, Vietnam; tranthebach.botany@gmail.com

**Keywords:** *Pavetta indica* L., triple-negative breast cancer cells, resistance, gas chromatography-mass spectrometry analysis, synergistic effect, 5,6-dehydrokawain

## Abstract

*Pavetta indica* L. is used in traditional medicine for the treatment of various diseases including hemorrhoids, headache, urinary conditions, ulcerated nose, and dropsy. However, no study has evaluated the anticancer effect of *P. indica* L. In this study, we found that a methanol extract of the leaves and branches of *P. indica* L. (MEPI) caused cell-cycle arrest at the sub-G1 phase and induced apoptosis, as indicated by the activation of caspase-8, -3, -7, and c-PARP. Western blotting revealed that MEPI significantly reduced the levels of markers of the epithelial-mesenchymal transition, such as Vimentin, Snail, Slug, and matrix metallopeptidase 9. Notably, the expression of multidrug resistance-associated protein 1 in triple negative breast cancer (TNBC) was significantly decreased by MEPI. Moreover, the co-treatment with MEPI and doxorubicin resulted in a synergistic reduction in cell viability. MEPI also induced radiation sensitization of TNBC cells. Gas chromatography-mass spectrometry analysis revealed that 5,6-dehydrokawain (DK) is the major constituent of MEPI. Interestingly, DK exerted significant anti-invasive and anti-metastatic effects. Our results provide a strong rationale for investigating the molecular mechanisms of action of MEPI in TNBC.

## 1. Introduction

*Pavetta indica* L. is a popular stout bushy shrub of the Rubiaceae family, distributed mainly in India, southern China, and northern Australia [1]. Parts of *P. indica* L. are used by traditional healers for the treatment of various diseases and conditions, including ulcerated nose, hemorrhoids [2,3], headache, urinary conditions, and dropsy [2]. *P. indica* L. reportedly exerted a hepatoprotective effect in a rat model of liver damage [4]. Moreover, a methanol extract of *P. indica* L. leaves exhibited anti-inflammatory activity in a rat model of inflammation [1]. However, the effect of *P. indica* L. methanol extract (MEPI) on cancer cells, including triple-negative breast cancer (TNBC) cells, is unclear.

According to the World Health Organization, breast cancer is the most common cause of cancer-related deaths among females worldwide. Among the subtypes of breast cancer, TNBC is the most aggressive, lacks the expression of estrogen receptor (ER), progesterone receptor (PR), and human epidermal growth factor receptor 2 (HER2), and accounts for 12–18% of all cases of breast cancer [5,6]. Hormone therapy is ineffective against triple-negative tumors due to their lack of PR, ER, and HER-2 [5]. Notably, TNBC has a high rate of resistance to chemotherapeutics due to the overexpression of epithelial–mesenchymal transition (EMT)-related factors [7] and drug transporters [8]. The epithelial–mesenchymal transition (EMT) is a biological process in which differentiated epithelial cells undergo molecular and morphological changes to become mesenchymal cells [9]. The EMT is characterized by the presence of mesenchymal markers (e.g., Vimentin, Snail, and Slug), and reduced levels of epithelial markers such as E-cadherin [10]. Following these morphological changes, the cancer cells become migratory and invasive due to an enhanced expression of matrix metallopeptidase 2 (MMP-2) and matrix metallopeptidase 9 (MMP-9) [11]. Induction of the EMT due to upregulation of the transcription factor transforming growth factor beta (TGF-β) causes epirubicin resistance in patients with TNBC [12]. ATP-binding cassette (ABC) drug transporters are transmembrane proteins that export a variety of substrates from the intracellular milieu, including therapeutic agents. In TNBC, the higher expression of intrinsic ABC transporters, such as breast cancer resistance protein (BCRP/ABCG2), multidrug resistance-associated protein 1 (MRP1/ABCC1), P-glycoprotein (P-gp/ABCB1), and multidrug resistance-associated protein 2 (MRP2/ABCC2), is associated with multidrug resistance and poor prognosis [8,13,14,15].

Surgery, chemotherapy, and radiotherapy are the only available treatment options for TNBC [16]. Resistance to chemo- and radio-therapy is a major limitation of cancer treatment. Doxorubicin (DOX) is a chemotherapeutic agent for TNBC that can induce apoptosis, senescence, and cell-cycle arrest at G1 in breast cancer cells [17,18]. However, the development of doxorubicin resistance can occur during treatment of patients with TNBC [19,20,21]. Thus, to overcome resistance in chemo- and radio-therapy, it is essential to develop new anticancer drugs or combinatorial drug regimens with increased efficacy and fewer side effects. Much effort has focused on developing novel anticancer drugs from natural sources, including plants [16,22].

The available preclinical evidence of the effect of *P. indica* L. on TNBC warrants investigation of the anticancer effects of a methanol extract of its leaves and branches (MEPI) on TNBC. We investigated the anticancer effect of MEPI on MDA-MB-231 TNBC cells by cell cycle analysis and viability, apoptosis, migration, and invasion assays. We found that MEPI exerted a synergistic effect with doxorubicin as well as radiation. Finally, gas chromatography-mass spectrometry (GC-MS) identified 5,6-dehydrokawain (DK) as the major compound in MEPI extract. These results suggest that MEPI has therapeutic potential in TNBC.

## 2. Results

### 2.1. MEPI Induced Apoptosis of MDA-MB-231 Cells

**Figure 1:**
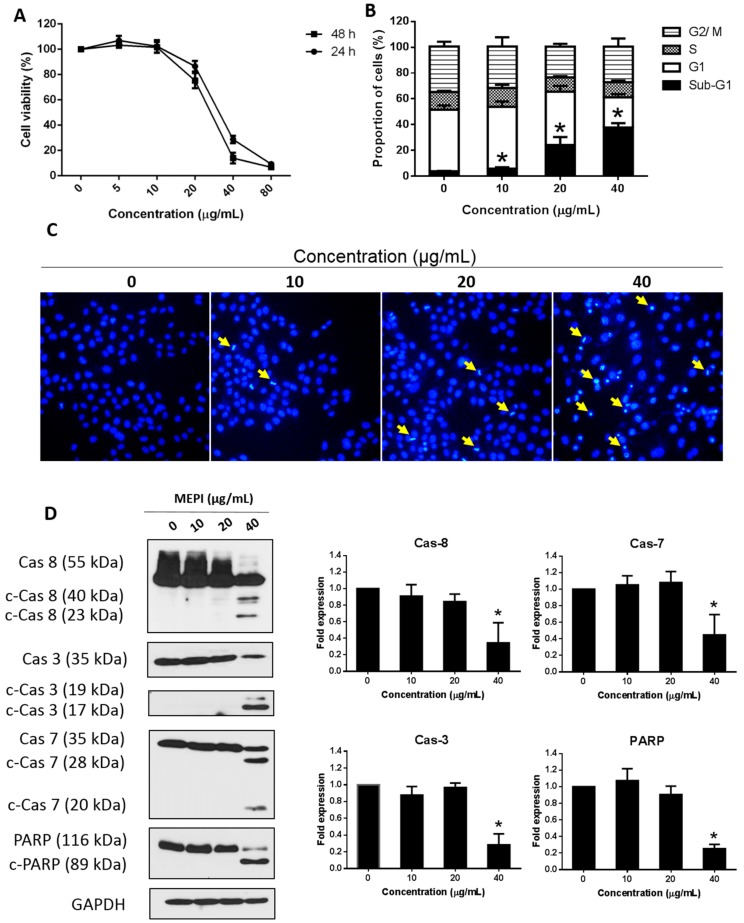
MEPI induced apoptosis in MDA-MB-231 cells. (**A**) Effect of MEPI on the growth of MDA-MB-231 cells at 24 and 48 h as determined by MTT assay. (**B**) Effect of MEPI on cell-cycle arrest in MDA-MB-231 cells. The cells were incubated with the indicated concentrations of MEPI for 24 h and the cell cycle distribution was assessed by flow cytometry with PI staining. (**C**) Cells stained with Hoechst 33,342 (100×). Nuclear fragmentation (yellow arrows) was observed by fluorescence microscopy following Hoechst 33,342 staining. (**D**) Western blot for caspase-8, c-caspase-8, caspase-7, c-caspase-7, caspase-3, c-caspase-3, PARP, and c-PARP after treatment with MEPI at the indicated concentrations for 24 h using Glyceraldehyde 3-phosphate dehydrogenase (GAPDH) as the internal control. Band intensities were measured using ImageJ software. Data are means ± standard deviation (SD). * *p* < 0.05.

We first examined the effect of MEPI (0–80 μg/mL for 24 or 48 h) on the viability of MDA-MB-231 cells by MTT assay (Figure 1A). MEPI exerted a cytotoxic effect on MDA-MB-231 cells, as indicated by IC_50_ values of 25.2 and 21.2 μg/mL at 24 and 48 h, respectively. Furthermore, flow cytometry with PI staining showed that the proportion of MDA-MB-231 cells at the sub-G1 phase was 3.74 ± 0.15% (DMSO only; 0 μg/mL MEPI) and 37.72 ± 1.94% (40 μg/mL MEPI) at 24 h, suggesting that MEPI induced cell-cycle arrest in MDA-MB-231 cells (Figure 1B). Next, to investigate whether the cytotoxicity of MEPI is mediated by the induction of apoptosis, Hoechst 33,342 staining was performed. Following treatment with MEPI for 24 h, nuclear fragmentation and chromatin condensation were evident in MDA-MB-231 cells (Figure 1C). Moreover, western blotting revealed that MEPI activated markers of apoptosis, including caspase-8, -3, -7, and c-PAPR, in a dose-dependent manner (Figure 1D). Taken together, these results indicate that MEPI induced cell cycle arrest at the sub-G1 phase and apoptosis in MDA-MB-231 cells.

### 2.2. MEPI Inhibited the EMT in MDA-MB-231 Cells

**Figure 2:**
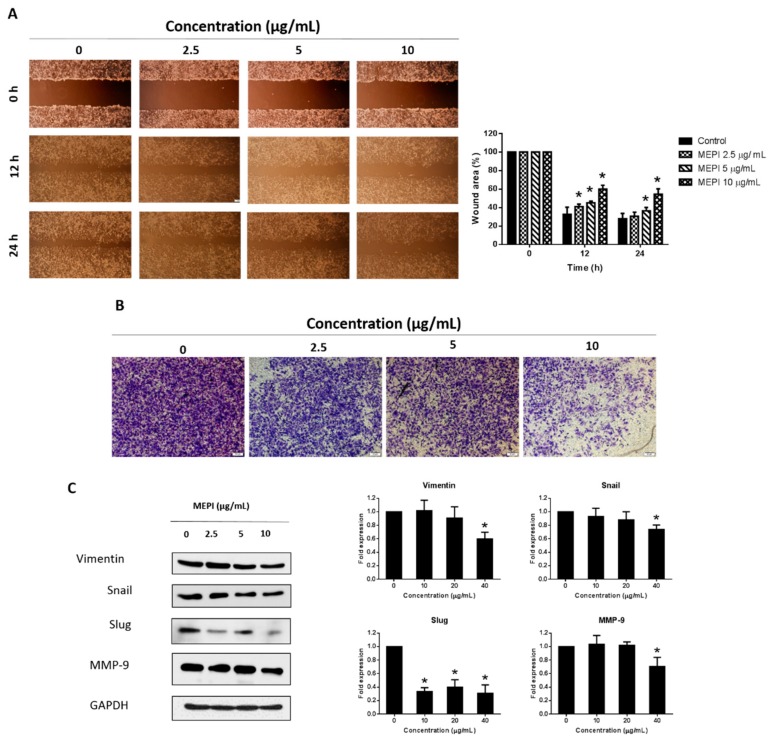
MEPI inhibited the epithelial–mesenchymal transition (EMT) in MDA-MB-231 cells. (**A**) Migration of MDA-MB-231 cells as determined by wound-healing assay. Wound area was calculated from a representative of at least three independent experiments. Percentages of wound closure at the indicated time points after MEPI treatment are shown. (**B**) Invasion of MDA-MB-231 cells. After incubation with MEPI for 24 h, cells that migrated through the Matrigel were stained with crystal violet and visualized by phase-contrast microscopy (40×). (**C**) Western blot for Vimentin, Snail, Slug, and MMP-9 after treatment for 24 h with MEPI at the indicated concentrations using GAPDH as the internal control. Band intensities were measured using ImageJ software. Data are means ± standard deviation (SD). * *p* < 0.05.

To assess the anti-metastatic effect of MEPI on MDA-MB-231 cells, we performed wound-healing and invasion assays. Treatment with 5 and 10 μg/mL MEPI resulted in a significant reduction in cell migration compared to the control (36.31 ± 1.40% and 54.44 ± 2.20%, respectively) (Figure 2A). Moreover, an invasion assay showed that the number of cells invading through the Matrigel was decreased by 5 and 10 μg/mL MEPI (Figure 2B). Consistent with these results, there was a decline in the levels of EMT-related proteins such as Vimentin, Snail, Slug, and MMP-9 (Figure 2C).

### 2.3. MEPI and Doxorubicin Synergistically Suppressed the Proliferation of MDA-MB-231 Cells

**Figure 3:**
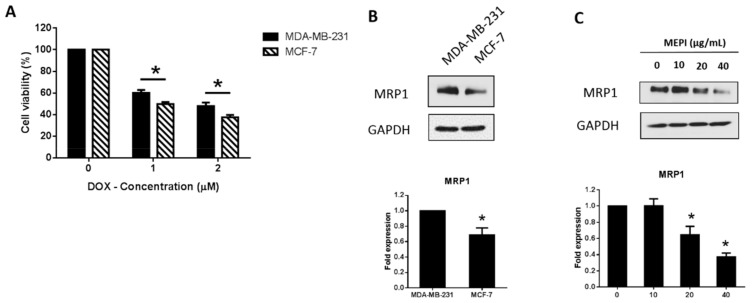
MEPI reduced the expression of MRP1 in MDA-MB-231 cells. (**A**) Effect of treatment with Doxorubicin (DOX) for 24 h on the viability of MDA-MB-231 and MCF-7 cells. (**B**) Western blotting for MRP1 in MDA-MB-231 and MCF-7 cells. (**C**) Western blotting for MRP1 at the indicated concentrations after treatment with MEPI for 24 h using GAPDH as the internal control. Band intensities were measured using ImageJ software. Data are means ± standard deviation (SD). * *p* < 0.05.

**Figure 4:**
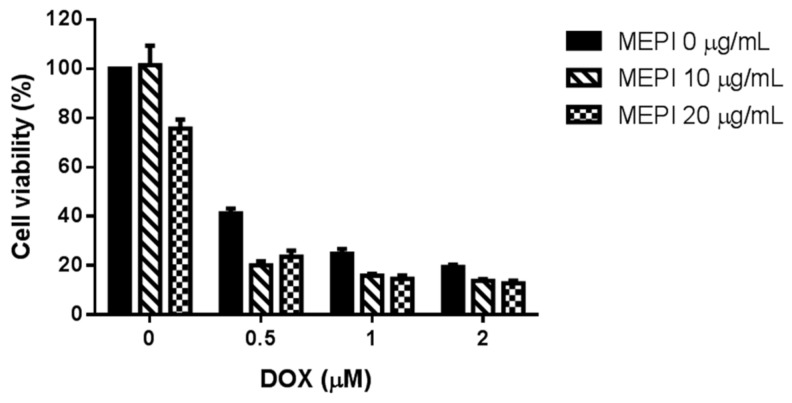
Co-treatment with MEPI and DOX at the indicated concentrations reduced the viability of MDA-MB-231 cells as determined by MTT assay. Data are means ± standard deviation (SD).

High expression of MRP1 is correlated with DOX resistance [23]. MDA-MB-231 cells were less sensitive to DOX treatment (1 and 2 μM) in comparison to MCF-7 cells (Figure 3A). Western blotting also revealed that the MRP1 level was higher in MDA-MB-231 cells than in MCF-7 cells (Figure 3B), and was reduced by MEPI (Figure 3C). To examine whether MEPI enhanced the cytotoxic effect of DOX, MDA-MB-231 cells were co-treated with MEPI (0–20 μg/mL) at the same time for 48 h with or without DOX (0–2 μM), and cell viability was assessed by MTT assay. Co-treatment with MEPI and DOX dramatically reduced the viability of MDA-MB-231 cells in comparison to DOX or MEPI alone (Figure 4). The combination index (CI) values confirmed that MEPI and DOX synergistically decreased the viability of MDA-MB-231 cells (CI < 1) (Table 1).

### 2.4. MEPI Induced Radiation Sensitization of MDA-MB-231 Cells

**Figure 5:**
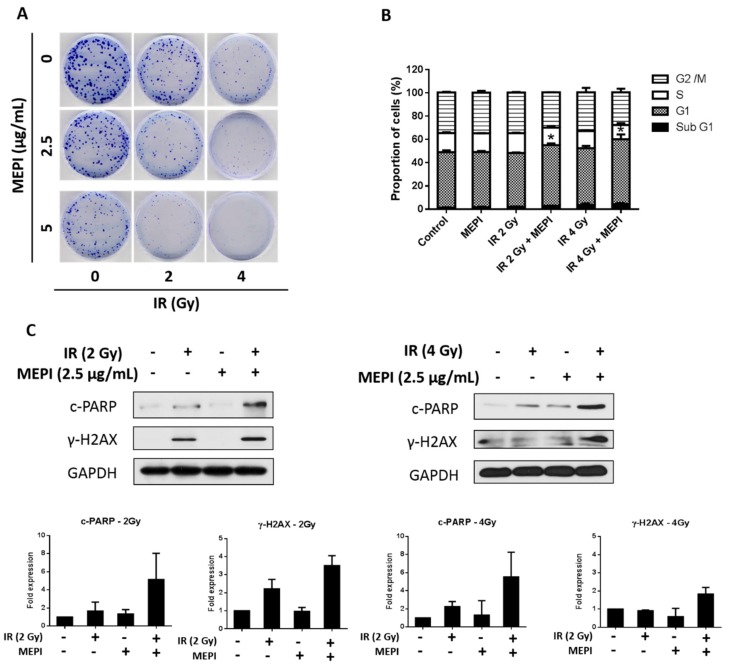
MEPI induced radiation sensitization of MDA-MB-231 cells. (**A**) Clonogenic assay after irradiation (2 or 4 Gy) and treatment with MEPI (2.5 or 5 μg/mL). (**B**) Analysis of the cell cycle after radiation (2 or 4 Gy) and treatment with MEPI (2.5 μg/mL). (**C**) Western blotting for c-PARP and γ-H2AX after irradiation (2 or 4 Gy) and treatment with MEPI (2.5 μg/mL). GAPDH was used as the internal control. Data are means ± standard deviation (SD). * *p* < 0.05.

We performed a colony formation assay to examine the responses of MDA-MB-231 cells to radiation therapy in the presence or absence of MEPI (2.5–20 μg/mL). MEPI enhanced the cytotoxicity induced by 2 or 4 Gy radiation compared to the radiation-only control (Figure 5A). PI staining revealed that radiation therapy together with MEPI at 2.5 μg/mL significantly induced cell-cycle arrest at the G1 stage in MDA-MB-231 cells compared to the radiation-only (2 or 4 Gy) control (Figure 5B). Indeed, western blotting showed that the co-treatment group had higher levels of c-PARP and γ-H2AX, biomarkers of DNA damage caused by radiotherapy (Figure 5C). Therefore, MEPI and radiation exerted a synergistic effect on MDA-MB-231 cells.

### 2.5. Chemical Composition of MEPI

**Figure 6:**
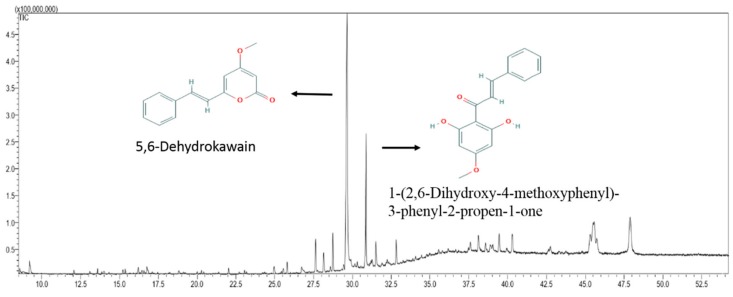
Gas chromatography-mass spectrometry (GC-MS) chromatogram of MEPI.

MEPI was analyzed by gas chromatography-mass spectrometry (GC-MS). The 12 compounds that were identified (Table 2), their chemical profile, and the total ion chromatogram (TIC) are shown in Figure 6. The dominant compound was identified as 5,6-dehydrokawain (66.65%), followed by 1-(2,6-dihydroxy-4-methoxyphenyl)-3-phenyl-2-propen-1-one (18.76%), stigmast-5-en-3-ol (3.78%), 6,11-dimethyl-2,6,10-dodecatrien-1-ol (2.83%), citral (2.25%), and phytol (1.43%). The other six compounds constituted less than 1% of the extract.

### 2.6. Determination of 5,6-Dehydrokawain in Pavetta Indica Methanol Extract

**Figure 7:**
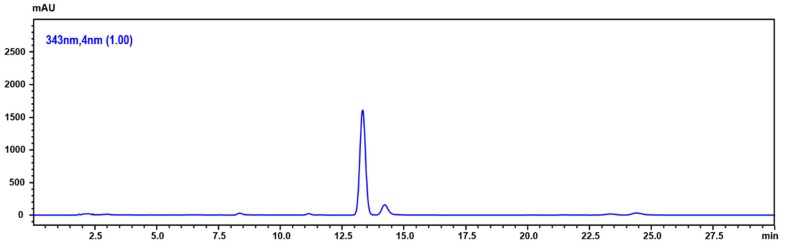
HPLC chromatogram of MEPI.

We confirmed the 5,6-dehydrokawain (DK), which is the major compound of MEPI from GC-MS analysis, by using the HPLC-DAD analysis. Figure 7 shows a chromatogram of the MEPI of DK at 343 nm. DK was detected at 13.68 min. The content of DK in this extract was 6.13 ± 0.76 mg/100 g.

### 2.7. 5,6-Dehydrokawain Inhibited the EMT in MDA-MB-231 Cells

**Figure 8:**
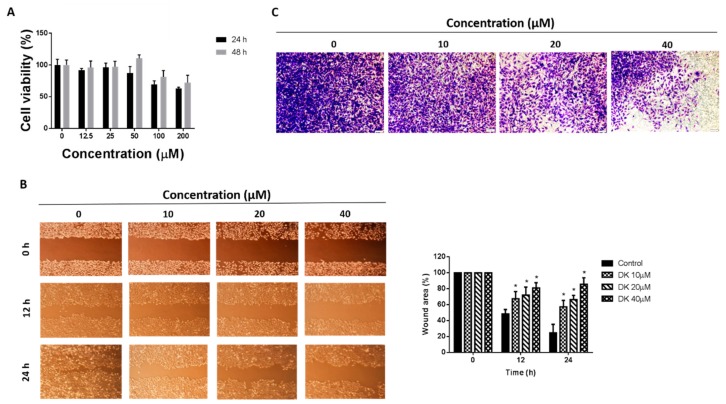
5,6-dehydrokawain (DK) inhibited the EMT of MDA-MB-231 cells. (**A**) Effect of treatment with DK for 24 and 48 h on the viability of MDA-MB-231 cells by MTT assay. (**B**) Migration of MDA-MB-231 cells determined by wound-healing assay. Wound area was calculated from a representative of at least three independent experiments. Percentages of wound area at the indicated time points after DK treatment are shown. (**C**) Invasion of MDA-MB-231 cells as determined by invasion assay. After incubation with DK at the indicated concentrations for 24 h, cells that migrated through the Matrigel were stained with crystal violet and visualized under a light microscope (40×). Data are means ± standard deviation (SD). * *p* < 0.05.

We next examined the anticancer effect of 5,6-dehydrokawain (DK), the major component of MEPI (Figure 6 and Figure 7, Table 2). The anti-proliferative activity of DK was determined by MTT assay (Figure 8A) and its anti-metastatic effect was determined by wound-healing and invasion assays using MDA-MB-231 cells. Although it did not exert a significant impact on the cell viability, DK at 10, 20, and 40 μM reduced the migration of MDA-MB-231 cells (57.25 ± 3.05, 66.31 ± 2.01% and 85.66 ± 3.10%, respectively) (Figure 8B). The percentage of invasive cells was decreased by treatment with DK at 20 and 40 μM (Figure 8C).

## 3. Discussion

Breast cancer is the major cause of cancer-related mortality among women worldwide. We report that MEPI exerted an anticancer effect on MDA-MB-231 TNBC cells. MEPI was cytotoxic to MDA-MB-231 cells (IC_50_ 25.2 μg/mL at 24 h) (Figure 1A). Also, PI staining of MDA-MB-231 cells treated with MEPI (20 and 40 μg/mL) for 24 h indicated that the cytotoxicity was mediated by cell cycle arrest at the sub-G1 phase (Figure 1B).

Apoptosis is a programmed cell death pathway induced by the activation of proteolytic enzymes known as caspases [24], which under normal conditions are present in their inactive forms. Following activation by cleavage, caspases activate downstream procaspases. Apoptosis may proceed by the extrinsic and intrinsic pathways. Whereas the intrinsic pathway is activated by intracellular signals, the extrinsic pathway is activated when an extracellular ligand binds to its cell-surface death receptor. Each pathway possesses its own specific initiator procaspases. For example, to activate the extrinsic apoptotic pathway, formation of the death-inducing signaling complex (DISC) complex by recruitment of procaspase-8 or -10 via adaptor proteins is required. This complex activates the cleavage of downstream caspases including caspase-3, -7, and -6, leading to the induction of apoptosis. In this study, MEPI reduced the expression of caspase-8, -7, and -3, suggesting the activation of the extrinsic apoptotic pathway in MDA-MB-231 cells (Figure 1D). Cleavage of PARP to c-PARP, which is mediated by caspase-3 and -7, is a marker of apoptosis [25]. We detected a decreased level of PARP and an increased level of c-PARP in MDA-MB-231 cells after treatment with MEPI (Figure 1D). Moreover, MEPI caused nuclear fragmentation and chromatin condensation, features of apoptotic cells (Figure 1C). Thus, MEPI induced caspase-dependent apoptosis of MDA-MB-231 cells.

The EMT is correlated with resistance to anticancer drugs [7,26] and its inhibition can overcome drug resistance [27]. The EMT is a biological process in which differentiated epithelial cells undergo molecular and morphological changes to become mesenchymal cells [9]. Compared to epithelial cells, EMT cells show increased invasiveness and migration [10]. The EMT is characterized by acquisition of mesenchymal markers (e.g., Vimentin, Snail, Slug, MMP-2, and MMP-9) and reduced levels of epithelial markers such as E-cadherin [10]. In this study, 10 μg/mL MEPI significantly reduced the migration and invasion (Figure 2A,B) of MDA-MB-231 cells and the levels of the EMT-related proteins Vimentin, Snail, Slug, and MMP-9 (Figure 2C).

ATP-binding cassette (ABC) drug transporters including MRP1 also mediate drug resistance by exporting therapeutic agents [8,13,14,15]. Doxorubicin (DOX) has been reported to enhance the expression of MRP1 in breast cancer cells, leading to resistance [28]. Additionally, in 2016, Chen et al. revealed that DOX significantly induced MRP1 expression levels in different non-small cell lung cancer cells (H1299, A549, and CH27 cells) in a time dependent manner [23]. It is reported that a high level of MRP1 is associated with a higher IC_50_ of DOX as well as a greater increase in DOX resistance due to the export of DOX [23]. In our study, in comparison with HER2-positive MCF-7 cells, MDA-MB-231 cells exhibited less sensitivity to DOX treatment at 1 and 2 μM (Figure 3A). Western blotting revealed that the expression of MRP1 was higher in MDA-MB-231 cells than in MCF-7 cells (Figure 3B), similar to the findings of Wan et al. [29]. The MRP1 level was decreased in a dose-dependent manner by treatment with MEPI for 24 h (Figure 3C).

Use of chemotherapeutics in combination with natural compounds can increase efficacy, reduce the dosage, minimize side effects, and overcome resistance [22]. Indeed, cucurbitacin B in combination with cisplatin synergistically inhibited the viability of H1975 and H820 cells [30]. Furthermore, Arctigenin has been reported to enhance the cytotoxicity of the conventional therapeutic agent, Taxotere^®^, in TNBC cells [31]. In this study, MEPI exerted a synergistic effect with DOX (Figure 4) in terms of reducing cell viability (CI < 1) (Table 1). MEPI also promoted the radiation sensitization of MDA-MB-231 cells by reducing colony formation and inducing cell-cycle arrest at the G1 phase (Figure 5A,B). Radiation therapy kills cancer cells by causing breaks in the DNA strands, which is indicated by the rapid phosphorylation of histone H2AX at serine 139 (γ-H2AX) [32] and cleavage of poly (ADP-ribose) polymerase (c-PARP) [33]. In this study, 2 or 4 Gy radiation induced DNA damage, as indicated by the induction of γ-H2AX and c-PARP, and the effect was enhanced by the co-treatment with 2.5 μg/mL MEPI (Figure 5C).

Moreover, previous studies revealed that the aroma compounds in herbs are associated with the active compounds and therapeutic effects [34] and the leaves of *Pavetta indica* L. are a good source of aromatic oils [35]. Therefore, we performed GC-MS and GC-MS derivatization (Supplementary Table S1 and Figure S1) to examine the compositions of MEPI. The results showed that there were totally 12 compounds identified in MEPI, with which the major constituent being DK (66.65%). DK has been isolated from several plant species; e.g., the *Alpinia speciosa* rhizome [36], *Alpinia mutica* Roxb. [37], *Polygonum hydropiper* [38], and *Alpinia zerumbet* [39]. Moreover, DK induces the production of the proangiogenic tumor-derived protein Vascular endothelial growth factor (VEGF) in colorectal cancer cells [37]; exerts anticholinesterase, antioxidant, and neuroprotective effects [40]; and promotes the differentiation of MC3T3-E1 osteoblasts [39]. In this study, the results of MTT, wound-healing, and invasion assays revealed that DK significantly decreased the migration and invasion of MDA-MB-231 cells (Figure 8A–C). Additionally, among the 12 compounds identified in MEPI, 1-(2,6-dihydroxy-4-methoxyphenyl)-3-phenyl-2-propen-1-one (18.76%), stigmast-5-en-3-ol (3.87%), citral (2.25%), and phytol (1.43%) reportedly have cytotoxic in vitro effects. 1-(2,6-dihydroxy-4-methoxyphenyl)-3-phenyl-2-propen-1-one, which is found in *Alpinia mutica* rhizomes, exerts a cytotoxic effect on different cancer cells, including breast cancer cells MCF-7 [41]. Fernando et al. found that stigmast-5-en-3-ol from *Dendronephthya gigantea* exerted proapoptotic and anti-proliferative effects on MCF-7 human breast cancer cells [42]. Additionally, citral has been shown to inhibit the proliferation of breast cancer [43] and stomach cancer [44] cells. Moreover, by reducing the expression of glutathione, a Reactive Oxygen Species (ROS) scavenger, citral activates apoptosis of breast cancer cells [43], and in combination with curcumin, induces their apoptosis and cell cycle arrest [45]. Furthermore, phytol inhibits the EMT in hepatocellular carcinoma cells [46].

In summary, MEPI induced apoptosis and cell cycle arrest at the sub-G1 phase, inhibited metastasis, and reduced the expression of MRP1 in MDA-MB-231 TNBC cells. Additionally, MEPI exerted a synergistic effect with DOX on, and induced radiation sensitization of, MDA-MB-231 cells by reducing colony formation and inducing cell cycle arrest at the G1 phase. Moreover, GC-MS analysis revealed that MEPI contained 12 compounds, among which DK, the major constituent, exerted anti-invasion and -migration effects. Consequently, MEPI shows therapeutic potential for TNBC.

## 4. Materials and Methods

### 4.1. Preparation of the P. indica Methanol Extracts

The *Pavetta indica* L. leaves and branches were obtained from the Korea Research Institute of Bioscience and Biotechnology (KRIBB, 125, Gwahak-ro, Yuseong-gu, Daejeon 34141, Korea, FBM074-099). The *Pavetta indica* L. leaves and branches (320 g) were mixed with 99.9% MeOH (16 L) and sonicated several times at room temperature for three days. The resulting MeOH extracts were filtered and evaporated at 40 °C under reduced pressure to afford crude extracts.

### 4.2. Cell Culture

The Human MDA-MB-231 cells and MCF-7 cells were obtained from the American Type Culture Collection (ATCC, Rockville, MD, USA) and cultured in Dulbecco’s modified Eagle’s medium (DMEM) supplemented with 10% heated-activated fetal bovine serum (FBS), 100 U/mL penicillin, and 100 μg/mL streptomycin. The cells were subcultured every three days and maintained in a humidified incubator at 37 °C with a 5% CO_2_ atmosphere.

### 4.3. Cell Viability Assay

The cells (2 × 10^4^/mL) were seeded in 96-well plates for 24 h and treated with MEPI, DOX (Sigma, St. Louis, MO, USA), or DK (Sigma, St. Louis, MO, USA) at the indicated concentrations. Next, the culture medium was replaced with 100 µL of fresh medium containing 0.5 mg/mL MTT and the plates were incubated for 2–3 h. The medium was aspirated from each well and 150 µL dimethyl sulfoxide (DMSO) were added to dissolve the formazan crystals. The absorbance at 570 nm was recorded using a microplate reader (Tecan Group, Ltd., Salzburg, Austria). The percentage cell viability was calculated using the formula (control group − treated group) ÷ control group) × 100% [47].

### 4.4. Flow Cytometric Analysis of the Cell-Cycle Distribution

The cells (3 × 10^4^/mL) were cultured in 60 mm dishes for 24 h and incubated with or without the indicated concentrations of MEPI for 24 h. Next, the cells were harvested, washed in phosphate-buffered saline (PBS), and fixed in 70% ethanol at −20 °C. The cells were washed in cold PBS, resuspended in 0.5 mL of PBS (2 mM EDTA) containing 50 mg/mL propidium iodide (PI) and 1 mg/mL RNase A, and incubated at 37 °C for 30 min. The stained cells were analyzed using a FACSCalibur flow cytometer (Becton Dickinson, Franklin Lakes, NJ, USA) at the wavelength of 550–700 nm.

### 4.5. Cell Migration Assay

The cells (1 × 10^5^/well) were seeded into six-well plates and cultured for 72 h. Next, scratches were made using a sterile 10 μL pipette tip and the cells were washed twice with PBS to remove debris. DMEM (5% FBS) with the indicated concentrations of MEPI was added, the plate was incubated for 24 h, and wound areas were photographed at 12-h intervals using an inverted phase-contrast microscope at 4× magnification.

### 4.6. Cell Invasion Assay

Cell invasion was evaluated using a Transwell system (24-well plate; Corning, Cambridge, MA, USA). First, 1.5 × 10^5^ MDA-MB-231 cells in 200 μL of serum-free medium supplemented with or without MEPI or DK were seeded in the upper chamber. The lower chamber was filled with 750 μL of DMEM supplemented with 10% FBS. After incubation for 24 h, the invading cells were fixed in formaldehyde followed by methanol and stained with 2% crystal violet. The stained cells were observed under a phase-contrast microscope [48].

### 4.7. Western Blot Analysis

Western blotting was performed as described previously [49]. Briefly, radioimmunoprecipitation assay lysis buffer was used to prepare cell lysates. The anti-GAPDH primary antibody (Cell Signaling Technology, Inc., Beverly, MA, USA) was used at a 1:7000 dilution and the other primary antibodies (Cell Signaling Technology, Inc., Beverly, MA, USA) were diluted in skim milk at a 1:1000 dilution. The anti-rabbit immunoglobulin G (IgG) secondary antibody (Vector Laboratories, Burlingame, CA, USA) was used at a 1:5000 dilution. The bands were developed using the BS ECL Plus Kit (Biosesang Inc., Seongnam, South Korea). The band intensities were measured using ImageJ software [50].

### 4.8. Analysis the Effects of Drug Combinations

The combination index (CI) values were calculated using CalcuSyn software (Biosoft, Ferguson, MO, USA). A CI value of <1, 1, or >1 indicates synergy, additivity, and antagonistic effects, respectively.

### 4.9. Gas Chromatography–Mass Spectrometry

GC-MS analysis was performed using Shimadzu GC-MS (Model QP-2010, Shimadzu Co., Kyoto, Japan) in the electron impact mode. The GC column was Rtx-5MS capillary column (30 m length, 0.25 mm internal diameter, and 0.25 µm film thickness). The injector temperature was set at 250 °C. The oven temperature was set at 80 °C (isothermal for 2 min), then ramped at 150 °C at 10 °C /min (isothermal for 1 min), then 220 °C at 5 °C /min (isothermal for 1 min), then 260 °C at 10 °C /min (isothermal for 0.5 min), and finally increased to 310 °C at 10 °C /min (isothermal for 20 min). Total GC-MS run time was 54.5 min, using helium as a carrier gas, at a flow rate of 1 mL/min. The methanol extract (2 mg) was dissolved into methanol (1 mL). The diluted samples of 1.0 µL were injected manually and the split ratio was 1:30. The Wiley 9th edited library data was used to search and identify each component, and to measure the relative percentage of each compound, relative peak areas of the total ionic chromatogram (TIC) were used, with calculations performed automatically.

### 4.10. Determination of 5,6-Dehydrokawain

The 5,6-dehydrokawain (chemical name 4-methoxy-6-[(*E*)-2-phenylethenyl]pyran-2-one, chemical formula C_14_H_12_O_3_, >98% purity) was purchased from abcam (Cambridge, UK). The 5,6-dehydrokawain was quantified using a high-performance liquid chromatography-diode array detector (HPLC-DAD; Shimadzu) equipped with a shim-pack GIS C18 column (250 × 4.6 mm, 5 μm ODS; Shimadzu), a quaternary pump, and an autosampler at 35 °C. The mobile phase eventually adopted for this study was acetonitrile/water (50/50), isocratic elution for 30 min and the flow rate was 1.0 mL/min. The sample injection volume was 10 μL. The detector was set to 343 nm. The peaks were identified by comparing their retention times and UV spectra with standards.

### 4.11. Irradiation

The cells (200/well) were seeded into 60 mm dishes and incubated for 24 h. Following treatment with MEPI (2.5 μg/mL), the cells were irradiated with 2 and 4 Gy of gamma rays at the Applied Radiological Science Institute at Jeju National University under the supervision of a radiologist. Ten days later, the colonies were washed twice with PBS, fixed with methanol, and stained with 2% crystal violet.

### 4.12. Statistical Analysis

The results were analyzed using GraphPad Prism version 6.01 for Windows (GraphPad Software, La Jolla, CA, USA), and are expressed as means ± standard deviation of three independent experiments. Student’s *t*-test was used to assess the significance of differences between the treated group and control group. The IC_50_ value was calculated by extrapolation.

## Figures and Tables

**Table 1:** Combination index (CI) values calculated by Calcusyn software.

MEPI (μg/mL)	DOX (μM)	CI Value
10	0.5	0.564
10	1	0.658
10	2	0.889
20	0.5	0.957
20	1	0.85
20	2	1.055

**Table 2:** Chemical profile of MEPI as determined by GC-MS analysis.

Peak No.	RT (min)	Compound Name	% Total	Mol. Formula	Mol. Wt (g/mol)
1	9.206	Citral	2.25	C_10_H_16_O	152.237
2	12.064	beta-Caryophyllene	0.68	C_15_H_24_	204.357
3	13.594	2,4-Di-tert-butylphenol	0.62	C_14_H_22_O	206.329
4	15.218	(-)-Spathulenol	0.53	C_15_H_24_O	220.356
5	15.362	Caryophyllene Oxide	0.73	C_15_H_24_O	220.356
6	16.208	Isospathulenol	0.97	C_15_H_24_O	220.356
7	22.024	Methyl palmitate	0.75	C_17_H_34_O_2_	270.457
8	25.795	Phytol	1.43	C_20_H_40_O	296.539
9	28.144	6,11-Dimethyl-2,6,10-dodecatrien-1-ol	2.85	C_14_H_24_O	208.34l
10	29.653	5,6-Dehydrokawain	66.65	C_14_H_12_O_3_	228.247
11	30.871	1-(2,6-Dihydroxy-4-methoxyphenyl)-3-phenyl-2-propen-1-one	18.76	C_16_H_14_O_4_	270.284
12	40.286	Stigmast-5-en-3-ol	3.78	C_29_H_50_O	414.718

RT, retention time; Mol., molecular; Wt., weight.

## Supplementary Materials

The following are available online, Figure S1: GC-MS derivatization of Pavetta Indica methanol extract, Table S1: Chemical profile of Pavetta Indica methanol extract using GC-MS derivatization.
