# Supplementary material for: Methanol Extract of Aerial Parts of Pavetta indica L. Enhances the Cytotoxic Effect of Doxorubicin and Induces Radiation Sensitization in MDA-MB-231 Triple-Negative Breast Cancer Cells
Source: Molecules. 2019 Jun 18;24(12):2273. doi: 10.3390/molecules24122273 (PMC6631732; doi:10.3390/molecules24122273)
Supplement: Supplementary file 1 [file molecules-24-02273-s001.pdf]

Article

Methanol Extract of Aerial Parts of *Pavetta indica* L. Enhances the Cytotoxic Effect of Doxorubicin and Induces Radiation Sensitization in MDA-MB-231 Triple-Negative Breast Cancer Cells

Yen Thi-Kim Nguyen [1,†], Jeong Yong Moon [2,†], Ji-yeon Ryu [3], Sangmi Eum [4], Tran The Bach [5] and Somi Kim Cho [1,2,3,*]

[1] Interdisciplinary Graduate Program in Advanced Convergence Technology and Science, Jeju National University, Jeju 63243, Korea; ntkyen.hcmus@gmail.com

[2] Subtropical/tropical organism gene bank, Jeju National University, Jeju 63243, Korea; owenmjy@jejunu.ac.kr

[3] School of Biomaterials Sciences and Technology, College of Applied Life Sciences, SARI, Jeju National University, Jeju 63243, Korea; rjo211@naver.com

[4] International Biological Material Research Center, Korea Research Institute of Bioscience & Biotechnology, 125, Gwahak-ro, Yuseong-gu, Daejeon 34141, Korea; sangeum@gmail.com

[5] Institute of Ecology and Biological Resources, Vietnam Academy of Science and Technology (VAST), Ha Noi 10000, Vietnam; tranthebach.botany@gmail.com

* Correspondence: somikim@jejunu.ac.kr; Tel.: +82-010-8660-1842

† These authors contributed equally to this work

Table S1. Chemical profile of *Pavetta Indica* methanol extract using GC-MS derivatization.

Peak No.	RT (min)	Compound name	% Total
1	6.467	Propanoic acid, 2-oxo-, trimethylsilyl ester	0.64
2	9.655	L-Alanine, trimethylsilyl ester	1.43
3	10.382	Glycerol, tris(trimethylsilyl) ether	1.63
4	16.091	.beta.-Alanine, N,N-bis(trimethylsilyl)-, trimethylsilyl ester	0.67
5	16.42	3,4-Bis(trimethylsilyloxy)butyric acid trimethylsilyl ester	0.33
6	19.411	Erythritol, tri-TMS	1.93
7	19.69	2-Pyrrolidone-5-carboxylic acid, trimethylsilyl ester	0.15
8	20.086	tert-Butyl(dimethyl)silyl 4-([tert-butyl(dimethyl)silyl]amino)butanoate	0.32
9	20.757	2,3,4-trihydroxybutyric acid tetrakis(trimethylsilyl) deriv. ((r* r*)-)	0.14
10	22.118	l-Proline, trimethylsilyl ester	0.41
11	23.858	ARABINO-1,5-LACTONE	0.13
12	27.512	Adonitol, pentakis(trimethylsilyl) ether	0.65
13	28.192	D-GLUCITOL, 1,2,3,4,5,6-HEXAKIS-O-(TRIMETHYLSILYL)-	0.59
14	29.064	D-Glucitol, 6-deoxy-1,2,3,4,5-pentakis-O-(trimethylsilyl)-	0.23
15	31.126	Shikimic acid tetrakis, trimethylsilyl ester	7.09
16	31.22	Citric acid, tetrakis-TMS	0.13
17	32.931	Quinic acid, trimethylsilyl ester	9.55
18	33.648	D-Fructose, 1,3,4,5,6-pentakis-O-(trimethylsilyl)-, O-methyloxime(TRIMETHYLSILYL)-	13.25
19	34.148	D-Fructose, 1,3,4,5,6-pentakis-O-(trimethylsilyl)-, O-methyloxime(TRIMETHYLSILYL)-	10.64
20	34.275	Galactose, trimethylsilyl ester	0.23
21	34.448	Galactose, trimethylsilyl ester	0.75
22	34.879	Mannose, MEOX-5TMS	14.69

23	35.526	GALACTOSE MEOX2 TMS	0.22
24	35.733	GALACTOSE MEOX1 TMS	2.29
25	36.561	D-Mannitol, TMS	2.69
26	36.915	D-GLUCITOL, 1,2,3,4,5,6-HEXAKIS-O-(TRIMETHYLSILYL)-	0.15
27	43.985	Palmitic Acid, TMS derivative	1.35
28	45.994	INOSITOL,O,O,O,O,O,O-TMS	6.69
29	46.6	RIBITOL-1,2,3,4,5-PENTATMS	0.16
30	47.206	GALACTOSE MEOX1 TMS	0.21
31	47.756	D-GLUCITOL, 1,2,3,4,5,6-HEXAKIS-O-(TRIMETHYLSILYL)-	0.15
32	51.463	Linoleic acid trimethylsilyl ester	0.46
33	51.763	trans-9-Octadecenoic acid, trimethylsilyl ester	1.29
34	52.908	Stearic acid, trimethylsilyl ester	0.54
35	56.834	D-GLUCITOL, 1,2,3,4,5,6-HEXAKIS-O-(TRIMETHYLSILYL)-	0.61
36	57.467	GLUCOHEPTULOSE MEOX1 TMS	0.94
37	57.785	Adonitol, pentakis(trimethylsilyl) ether	1.08
38	65.325	SUCROSE-OCTATMS	8.46
39	66.598	MALTOSE MEOX2 TMS	0.83
40	67.332	6,7-DIHYDROXYCOUMARIN-.BETA.-D-GLUCOPYRANOSIDE, PENTA-TMS	0.67
41	67.75	MALTOSE-OCTATMS	0.45
42	67.989	TREHALOSE TMS	0.75
43	68.136	MALTOSE MEOX2 TMS	0.39
44	69.221	MALTOSE MEOX2 TMS	0.78
45	70.225	MALTOSE MEOX2 TMS	0.19
46	70.5	Nonadecanoic acid, trimethylsilyl ester	0.44
47	71.149	MALTOSE MEOX2 TMS	0.74
48	73.817	Galactose, trimethylsilyl ether	0.44
49	79.438	METHYL 18-HYDROXYOCTADECA-9,12-DIENOATE TMS ETHER	0.47
50	79.981	Stigmasterol, trimethylsilyl ether	0.42
51	81.242	Campesterol, trimethylsilyl ether	0.56

(1)

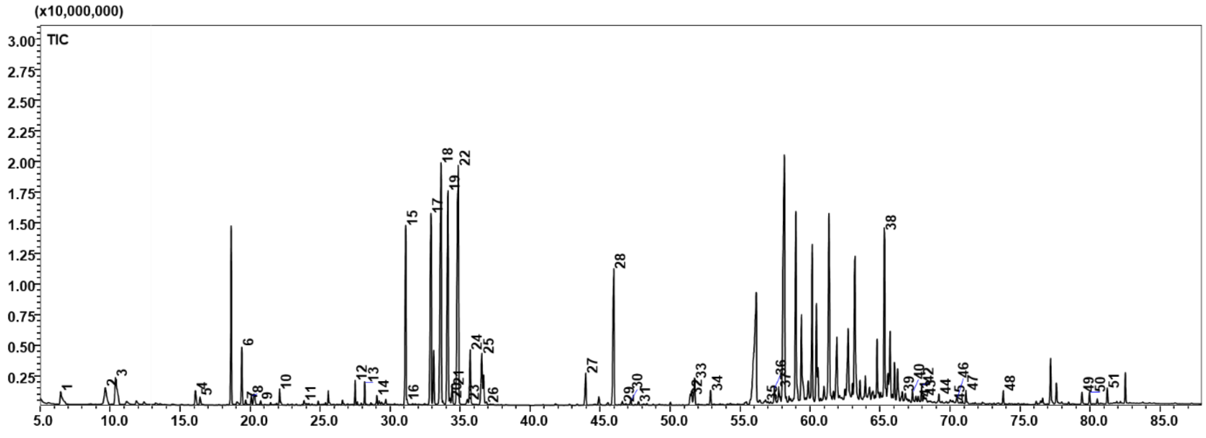

Figure 1. GC-MS derivatization of *Pavetta Indica* methanol extract.
